# Partial Splenic Embolization with Transarterial Chemoembolization in Patients with Hepatocellular Carcinoma Accompanied by Thrombocytopenia

**DOI:** 10.1155/2014/960628

**Published:** 2014-09-15

**Authors:** Yoshihiko Ooka, Tetsuhiro Chiba, Sadahisa Ogasawara, Tenyu Motoyama, Eiichiro Suzuki, Akinobu Tawada, Fumihiko Kanai, Osamu Yokosuka

**Affiliations:** Department of Gastroenterology and Nephrology, Graduate School of Medicine, Chiba University, 1-8-1 Inohana, Chuo-ku, Chiba 260-8670, Japan

## Abstract

*Background*. Thrombocytopenia often makes the introduction of systemic treatment difficult in patients with cirrhosis and hepatocellular carcinoma (HCC). We retrospectively evaluated the long-term effects of partial splenic embolization (PSE) with transarterial chemoembolization (TACE) in patients with HCC patients accompanied by thrombocytopenia. *Patients and Methods*. Twenty-one patients with HCC complicated by severe thrombocytopenia (platelet count, <5.0 × 10^4^/mm^3^) were treated with PSE and TACE. Both the safety and platelet-increasing effect was evaluated in these patients. *Results*. Seventeen of 21 patients (81.0%) showed increased platelet counts to ≥5.0 × 10^4^/mm^3^. Subsequently, 13 patients (61.9%) successfully received systemic chemotherapy. Platelet counts and serum levels of total bilirubin, as well as neutrophil counts, improved significantly one month after treatment. However, serum levels of albumin and hemoglobin decreased significantly one month after treatment. Severe adverse events, including acute liver failure and portal vein thrombus, were observed in two patients. *Conclusion*. PSE with selective TACE made it possible for patients with HCC and severe thrombocytopenia to receive systemic chemotherapy. Although PSE with TACE was safe and tolerable for most patients, the extent of PSE with TACE in a wide area of the liver may increase the risk for fatal liver failure.

## 1. Introduction

Hepatocellular carcinoma (HCC) frequently develops in patients with cirrhosis. A considerable number of patients with HCC have thrombocytopenia secondary to hypersplenism. Since cirrhotic patients with severe thrombocytopenia are at greater risk for bleeding, treatments against HCC such as liver transplantation, resection, local ablation therapy, and transarterial chemoembolization (TACE) can be performed following a platelet transfusion in those patients. Therefore, there have been minimal differences in treatment strategy between patients with or without severe thrombocytopenia.

Sorafenib has shown survival benefit in patients with advanced HCC patients [1, 2]. However, sorafenib has several side effects such as decreased platelet counts and bleeding. Therefore, patients with severe thrombocytopenia are difficult to treat with sorafenib, and their treatment options are limited; they can only receive repetitive TACE or the best supportive treatment. These findings indicate that a new strategy to treat patients with advanced HCC and severe thrombocytopenia is needed.

Over the past 20 years, partial splenic embolization (PSE) has been an interventional radiological treatment for thrombocytopenia due to hypersplenism [3, 4]. This procedure usually results in an increase of platelet count. Recent reports show that PSE has a platelet-increasing effect and allows for the introduction of interferon therapy or systemic chemotherapy [5–8]. Therefore, we considered that PSE and TACE should be performed simultaneously to allow the introduction of systemic chemotherapy. The aim of this study was to assess the safety and effectiveness of PSE with TACE for patients with HCC and severe thrombocytopenia.

## 2. Patients and Methods

### 2.1. Patients

Medical records were retrieved for patients with HCC who were treated by PSE with TACE from November 2010 to October 2012 at Chiba University Hospital. Patients were included if they met the following criteria: (i) no indication for surgical resection or locoregional therapy, (ii) severe thrombocytopenia (platelet count, <5.0 × 10^4^/mm^3^), (iii) Child-Pugh A or B, (iv) Eastern Cooperative Oncology Group (ECOG) performance status 0 or 1, and (v) follow-up dynamic computed tomography (CT) 1 week after PSE with TACE. This study was approved by the Research Ethics Committee of Graduate School of Medicine, Chiba University.

### 2.2. PSE and TACE Procedure

All patients received sedative premedication with intravenous pentazocine and hydroxyzine. The procedure was performed under the local anesthesia using 1% xylocaine. A femoral artery approach was used for catheterization. Patients underwent baseline angiography of the celiac trunk, superior mesenteric artery, hepatic artery, and splenic artery using a peripheral arterial approach. First, we performed PSE according to Takatsuka method [9]. We placed a 4-Fr catheter into the splenic artery as near the splenic hilum as possible. A 2.2-, 2.4-, or 2.7-Fr microcatheter was coaxially advanced as distally as possible into a branch of the intrasplenic artery. We left the microcoils straight in the branch of the splenic artery. About 5-mm sized gelatin sponges were implanted proximal to the microcoils. Lower branches of the splenic artery were embolized using 0.035 inch coils (Tornado or Nester; Cook Inc., Bloomington, IN, USA). The length of coils was determined by that of the targeted arteries. It has been recommended that the final embolization rate of spleen is about 70% [9]. However, giving priority to the safety of patients, this procedure was repeated until a splenic infarction of about 40% to 60% was achieved, as shown on digital subtraction angiography. After PSE procedure was completed, we performed the TACE. Highly selective catheterization was performed with a microcatheter to obtain complete obstruction of the nourishing arteries and avoid damage to nontumor tissue. Cisplatin or epirubicin was emulsified with lipiodol and miriplatin was suspended with lipiodol. These were administered before embolization. Additional Gelpart (Nippon Kayaku Co., Ltd., Tokyo, Japan) was used to complete the embolization procedure. Lipiodol CT was performed at the end of the procedure to confirm the embolized area in the liver. Methylprednisolone (125 mg intravenously for 1–3 days) and naproxen (400 mg per oral for 1 week) were given to relieve postembolic syndrome. Antibiotic prophylaxis was given for 1 week to avoid infectious complications.

### 2.3. Follow-Up

Dynamic CT was performed in all patients 1 week after the procedure to confirm the area of splenic infarction. When a patient with extrahepatic progression successfully showed an increase in platelet counts (≥5.0 × 10^4^/mm^3^) 1 month after PSE with TACE, systemic chemotherapy was started. Follow-up dynamic CT was performed every 2-3 months. In patients without extrahepatic progression, dynamic CT was performed every 3-4 months after PSE with TACE to evaluate the therapeutic effect of TACE. When patients showed status that met the criteria proposed by the 2010 Japan Society of Hepatology Consensus Guidelines for TACE-refractoriness [10] and an increase in platelet counts (≥5.0 × 10^4^/mm^3^), systemic chemotherapy was started. In patients with HCC controlled by TACE, re-TACE with PSE was performed when HCC progressed. In patients with platelet counts sustained <5.0 × 10^4^/mm^3^, re-TACE with PSE was performed until it could not be continued.

### 2.4. Calculation of Splenic Volume and Noninfarcted Splenic Volume

Splenic and noninfarcted splenic volumes in each patient were calculated using a three-dimensional workstation (Zio Station, Zio Software Inc., Tokyo, Japan). We used volume data from a portal phase multidetector row CT scan taken before and 1 week after the procedure. We performed the masking manually so that only the enhanced spleen remained on the workstation. Masking volume was calculated automatically by the workstation and we made it noninfarcted splenic volume.

### 2.5. Assessment of Adverse Events

We calculate the rate of adverse events in total of 32 PSE with TACE procedures in 21 patients to evaluate safety. Adverse events were evaluated according to the National Cancer institute Common Terminology Criteria for Adverse Events (CTCAE) ver. 4.0 (URL: http://ctep.cancer.gov/protocolDevelopment/electronic_applications/ctc.htm#ctc_40).

### 2.6. Statistical Analysis

The change of neutrocyte counts, platelet counts, hemoglobin, albumin, prothrombin time-international normalized ratio (PT-INR), total bilirubin, and Child-Pugh score after PSE with TACE was evaluated before and after PSE with TACE. The paired* t*-test with a two-tailed test was used to compare between the data before and after PSE with TACE. All statistical analyses were performed using SPSS software (IBM SPSS Statistics 18; SPSS Japan).

## 3. Results

### 3.1. Patient Characteristics and Outcomes

**Figure 1:**
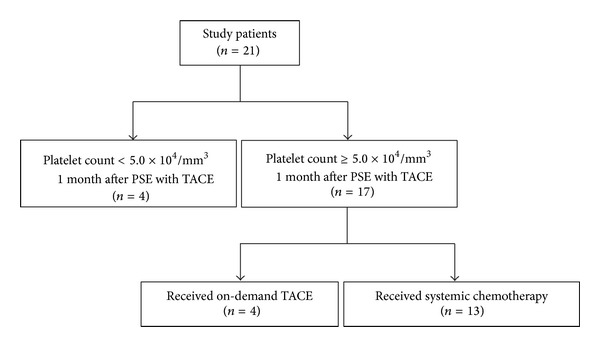
Patient outcomes.

Twenty-one patients (20 males and one female; mean age, 63.0 ± 9.1) with HCC and severe thrombocytopenia received PSE with TACE.  Table 1 shows the characteristics of the 21 patients. The causes of chronic liver damage were hepatitis C virus (HCV) (*n* = 14), hepatitis B virus (HBV) (*n* = 3), and non-HBV and non-HCV (*n* = 4). Seventeen patients were in Barcelona Clinic Liver Cancer- (BCLC-) B stage and four patients were in BCLC-C stage. Mean splenic volume before PSE was 630.5 ± 239.0 mL. The mean platelet count before treatment was 4.2 ± 0.7 × 10^4^/mL. Seventeen patients showed an increase in platelet counts ≥5.0 × 10^4^/mm^3^after PSE with TACE for 1 month, although the platelet counts in four patients remained <5.0 × 10^4^/mm^3^ (Figure 1). Among them, one died within 1 month because of liver failure, one showed portal vein thrombosis, one was lost to follow-up, and one exhibited totally no effect despite two additional PSE procedures. Four of 17 patients showed a successful increase in platelet counts and a simultaneous anti-HCC effect by TACE. Thereafter, they received on-demand TACE but not systemic chemotherapy. Eventually, 13 of 21 patients (61.9%) received systemic chemotherapy after first PSE with TACE. Notably, no patients withdrew or decreased the dose of their systemic chemotherapy because of thrombocytopenia. Four patients, who failed first-time systemic chemotherapy, received additional TACE with PSE before second-line systemic chemotherapy. One patient had variceal bleeding and received endoscopic variceal ligation and injection sclerotherapy before PSE and TACE. The 3 patients who experienced variceal bleeding after the procedure had severe liver dysfunction caused by HCC progression or were receiving sorafenib. Therefore, a causal connection between PSE and variceal bleeding was unclear.

### 3.2. Changes in Laboratory Data

Table 2 shows the changes in the neutrocyte count, platelet count, hemoglobin level, albumin level, PT-INR, total bilirubin level, and Child-Pugh score before treatment, one week later and one month later. The neutrocyte count increased significantly 1 week after treatment (*P* = 0.001). The albumin and hemoglobin levels decreased significantly 1 week after treatment (*P* < 0.001 and *P* = 0.012, resp.). Child-Pugh score deteriorated significantly 1 week after treatment (*P* = 0.021). The platelet count, total bilirubin level, and neutrocyte count improved significantly 1 month after treatment (*P* < 0.001, *P* = 0.039, and *P* < 0.001, resp.). Although the albumin and hemoglobin levels decreased significantly 1 month after treatment (*P* = 0.036 and *P* = 0.046, resp.), they improved compared with those of 1 week after treatment. However, neither PT-INR nor the Child-Pugh score changed significantly between before and after treatment.

### 3.3. Adverse Events

Major adverse events observed in 32 PSE with TACE procedures (in 21 patients) were shown in Table 3. Thirteen patients underwent the procedure once, 5 underwent the procedure twice, and 3 underwent the procedure thrice. In >30% procedures, increased levels of aspartate aminotransferase (AST)/alanine aminotransferase (ALT), pain, ascites, and increased level of blood bilirubin were observed. Most of these adverse events improved without treatment or with only conservative treatment. PSE often causes severe complications such as splenic abscess, splenic rupture, pneumonia, refractory ascites, and pleural effusion [11, 12]. Neither splenic rupture nor pneumonia was observed in this study. Although splenic abscess was observed in one patient, he was improved by systemic administration of antibiotics. Although fourteen and 5 patients exhibited ascites and pleural effusion, respectively, these complications were temporary but not refractory.

One patient died because of liver failure. He had multiple HCCs that occupied >50% of the liver and underwent PSE with TACE in a wide area of the liver. One patient developed portal vein thrombosis. A reevaluation of the dynamic CT images revealed a mural thrombus in the portal vein before treatment. The thrombus resolved following warfarin administration.

## 4. Discussion

PSE is a useful procedure to improve hypersplenism and portal hypertension and their complications [13–18]. However, there are only a few reports that are available about PSE in patients with HCC [19–21]. In those reports, PSE with TACE was mainly performed to decrease complications of portal hypertension such as variceal bleeding. However, endoscopic therapy is the method most commonly used to prevent variceal bleeding. Considering that PSE has several severe complications [13, 22], the advantage of PSE was relatively small with regard to the treatment of gastroesophageal varices. Systemic chemotherapy using anticancer agents such as sorafenib is now available for patients with advanced HCC. However, patients with severe thrombocytopenia, one of the complications of hypersplenism, cannot be treated with systemic chemotherapy because of chemotherapy-induced platelet-decreasing effect. Improving the severe thrombocytopenia enables those patients to receive systemic chemotherapy. No reports have considered the use of PSE to introduce systemic chemotherapy to patients with advanced HCC and severe thrombocytopenia. Thus, we evaluated the efficacy and safety of PSE for the purpose of introducing systemic chemotherapy to patients with HCC.

Our treatment strategy for patients with HCC and severe thrombocytopenia was simultaneous PSE and TACE. If the first procedure did not achieve the goal, we repeated the procedure when HCC progressed. This strategy has several advantages. First, if PSE does not achieve the purpose in the first session, patients undergo TACE. Second, thrombocytopenia may improve as well as portal hypertension, which prevents other complications. Our results showed that >80% of patients demonstrated an increase in platelet counts after PSE with TACE. In addition, approximately 60% of patients successfully received systemic chemotherapy for HCC. It has been reported that recurrence of thrombocytopenia was observed in some cases treated with PSE [3, 4]. Because more than half of the patients received sorafenib administration within one year after PSE in this study, continuous effect of PSE against thrombocytopenia remains undetermined. However, no patients withdrew or decreased the dose of their systemic chemotherapy because of thrombocytopenia. Taken together, these results indicate that TACE with PSE is an effective method for patients with HCC and severe thrombocytopenia.

Most of adverse events which occurred after PSE with TACE were temporary and controllable. However, severe adverse events including portal vein thrombosis and liver failure occurred in two patients. One patient was complicated with portal vein thrombosis, which was successfully treated with warfarin, and the thrombus resolved gradually. The other patient, who had been treated with PSE and TACE in a wide area in the liver, died because of liver failure. Considering that the extent of splenic infarcted area after PSE was relatively high (77.0%), not only TACE but also PSE may have been involved in liver failure. Previous reports demonstrated that severe complications of PSE often develop when the extent of infarcted area is >70.0% [13, 21, 22]. Therefore, liver failure might develop after PSE even without simultaneous TACE in this case. Taken together, the PSE treatment area should be <70.0% at one session in order to prevent severe complications. This study had several limitations. First, patient population was small. Second, our study had only one arm and we did not compare patients who were treated with only TACE. Further studies are needed to clarify the efficacy of PSE with TACE.

## 5. Conclusion

In conclusion, PSE for patients with HCC severe thrombocytopenia was useful to increase platelet counts and made it possible to introduce systemic chemotherapy. PSE with selective TACE is tolerable in most cases, but the extent of PSE with TACE in a wide area of the liver may increase the risk for fatal liver failure.

## Figures and Tables

**Table 1:** Patient data.

Number	Age	Gender	Etiology	BCLC	C-P score	Splenic volume (mL)	Extent of necrosis (%)	Platelet count (×10^4^/mm^3^)		Hospital time (days)	Introduction of SC
								Before	After 1 month		
1	48	M	HCV	B	5	578.4	45.3	4.8	11.7	11	Success
2	60	M	HCV	B	5	990.5	38.9	3.9	6.0	10	Success
3^a^	64	M	NBNC	B	7	1116.6	39.1	3.9	5.6	11	Failure
4	57	M	HCV	B	5	528.3	40.6	2.7	4.3	13	Failure
5^b^	65	M	HCV	B	7	426.3	36.3	2.4	ND	12	Failure
6	48	M	HBV	C	6	948.1	68.5	4.8	17.5	20	Success
7	63	M	HCV	B	6	656.7	72.5	4.6	15.1	15	Success
8	78	F	HCV	C	5	727.0	42.7	3.7	6.0	9	Success
9	55	M	HCV	B	5	401.3	19.5	4.9	7.2	12	Success
10	82	M	NBNC	B	8	472.4	40.2	4.8	8.7	9	NA
11	55	M	HBV	B	7	896.8	21.0	4.1	6.1	8	NA
12^c^	66	M	HCV	B	7	815.9	30.2	4.5	4.6	12	NA
13	54	M	NBNC	B	5	469.5	23.5	4.6	13.1	11	Success
14	64	M	HBV	B	5	469.5	20.1	4.8	13.7	9	Success
15	64	M	HCV	B	5	997.7	31.1	3.9	9.4	10	Success
16	61	M	HCV	B	6	411.9	47.7	4.6	7.3	9	NA
17	76	M	HCV	B	6	287.7	51.2	4.4	7.9	12	Success
18	53	M	HCV	C	6	270.3	81.6	4.9	15.4	8	Success
19^d^	65	M	HCV	B	8	785.5	77.0	3.4	ND	17	Failure
20	68	M	NBNC	C	6	491.1	30.1	3.4	8.7	20	Success
21	77	M	HCV	B	6	499.6	36.0	4.8	7.7	10	Success

Mean	63.0 ± 9.1					630.5 ± 239.0	42.5 ± 17.6	4.2 ± 0.7	9.3 ± 3.8	11.4 ± 3.5	

^a^Complicated by portal vein thrombosis, ^b^lost to follow-up, ^c^no effect despite two additional PSE, ^d^complicated by lethal liver failure; BCLC, Barcelona Clinical Liver Center; C-P, Child-Pugh; SC, systemic chemotherapy; HBV, hepatitis B virus; HCV, hepatitis C virus; NBNC, non-HBV and non-HCV; PSE, partial splenic embolization; ND, not determined; NA, not applicable.

**Table 2:** Changes of the laboratory data between before treatment and 1 month after partial splenic embolization with transarterial chemoembolization.

	Pretreatment	After 1 week			After 1 month		
	Data	Data	Mean of difference	*P* value	Data	Mean of difference	*P* value
Platelet counts (×10^4^/mm^3^)	4.3 ± 0.6	5.6 ± 2.1	0.9 (−0.1 to 2.0)	0.083	9.3 ± 4.0	4.9 (3.2 to 6.7)	<0.001
Hemoglobin (g/dL)	12.3 ± 1.8	11.5 ± 2.0	−0.7 (−1.2 to 0.2)	0.012	11.8 ± 1.4	−0.5 (−1.0 to −0.0)	0.046
Neutrocyte counts (/mm^3^)	1,878 ± 851	4,269 ± 2,797	2,241 (1,016 to 3,047)	0.001	2,565 ± 1,055	687 (408 to 965)	<0.001
Total bilirubin (mg/dL)	1.5 ± 0.8	1.7 ± 0.8	0.2 (0.0 to 0.5)	0.063	1.3 ± 0.6	−0.2 (−0.4 to 0.0)	0.039
Albumin (g/dL)	3.6 ± 0.2	3.2 ± 0.4	−0.4 (−0.6 to −0.3)	<0.001	3.5 ± 0.3	−0.1 (−0.3 to 0.0)	0.036
PT-INR	1.16 ± 0.14	1.21 ± 0.21	0.03 (−0.01 to 0.07)	0.123	1.12 ± 0.14	−0.04 (−0.08 to 0.01)	0.092
Child-Pugh score	5.8 ± 0.9	6.3 ± 0.7	0.5 (0.1 to 0.9)	0.021	5.9 ± 0.7	0.1 (−0.3 to 0.4)	0.748

PT-INR, prothrombin time-international normalized ratio.

**Table 3:** Adverse events.

		*n* = 32
	Any grade number (%)	≥grade 3 number (%)
Increased AST/ALT levels	24 (75)	9 (28)
Pain	19 (59)	0
Increased ALP level	15 (46)	0
Ascites	14 (44)	0
Increased bilirubin level	11 (34)	2 (6)
Anorexia	6 (19)	0
Pleural effusion	5 (16)	0
Fever	4 (13)	0
Decreased leukocyte counts	3 (9)	2 (6)
Increased PT-INR	3 (9)	2 (6)
Hyperkalemia	3 (9)	1 (3)
Hypocalcemia	3 (9)	0
Constipation	3 (9)	0
Increased CPK level	3 (9)	0
Decreased neutrocyte counts	2 (6)	2 (6)
Hyperuricemia	2 (6)	1 (3)
Nausea	2 (6)	0
Diarrhea	2 (6)	0
Hematoma	2 (6)	0
Increased creatinine level	2 (6)	0
Hepatic failure	1 (3)	1 (3)
Portal vein thrombosis	1 (3)	1 (3)
Splenic abscess	1 (3)	0
Splenic rupture	0	0
Pneumonia	0	0

AST, aspartate aminotransferase; ALT, alanine aminotransferase; ALP, alkaline phosphatase; PT-INR, prothrombin time-international normalized ratio; CPK, creatine phosphokinase.
